# Level of and Factors Determining Physical Activity in Students in Ukraine and the Visegrad Countries

**DOI:** 10.3390/ijerph15081738

**Published:** 2018-08-13

**Authors:** Józef Bergier, Anatolii Tsos, Dariya Popovych, Barbara Bergier, Ewelina Niźnikowska, Pongrác Ács, Jan Junger, Ferdinand Salonna

**Affiliations:** 1 Pope John Paul II State School of Higher Education, 21-500 Biala Podlaska, Poland; barbara.bergier@wp.pl (B.B.); rewela.n@op.pl (E.N.); 2 Institute of Physical Education and Health, Lesya Ukrainka Eastern European National University, 43025 Lutsk, Ukraine; Tsos.Anatoliy@eenu.edu.ua; 3 Department of Physical Rehabilitation, Human Health and Physical Education, I. Horbachevsky Ternopil State Medical University, 46001 Ternopil, Ukraine; darakoz@yahoo.com; 4 Institute of Physiotherapy and Sport Science, University of Pécs, H-7623 Pécs, Hungary; pongrac.acs@etk.pte.hu; 5 Pavol Jozef Šafárik University in Kosice, Institute of Physical Education and Sport, 041 80 Košice, Slovak; jan.junger@upjs.sk; 6 Faculty of Physical Culture, Palacky University of Olomunec, 771 47 Olomouc, The Czech Republic; ferdinand.salonna@upol.cz

**Keywords:** physical activity, IPAQ, students, BMI, self-assessment of physical fitness, leisure time, gender

## Abstract

*Background*: Examining the level of physical activity in students is a vital issue as these young people are the future social elite. *Methods*: The research was conducted in the years 2013–2016 and comprised 5008 males and females, mean age of 20.5 ± 2.1 years, including 2237 (55.3%) students from students from Eastern European National University, Lutsk, and Ternopil State Medical University, Ukraine, as well as 2237 (44.7%) from the Visegrad countries, i.e., University of Pécs in Hungary, University in Kosice, Slovakia, University of Olomunec in the Czech Republic and the State School of Higher Education in Biała Podlaska, Poland. It applied a diagnostic survey—the extended version of the International Physical Activity Questionnaire (IPAQ), supplemented with original questions regarding body weight, height, self-assessment of physical fitness and amount of leisure time. *Results*: The comparison of students’ PA from selected European countries, members of the Visegrad group and Ukraine, is particularly vital to the latter country as it demonstrates a different level of socio-economic development. There was noted a significantly higher level of physical activity in students from the Visegrad countries than in Ukraine. Further, there was an overall lower level of physical activity in females compared to males. What was positive about the studied samples was a healthy BMI index in the majority of the male and female respondents, with a significantly higher indicator in Ukraine. *Conclusions*: Among the factors significantly affecting higher physical activity in both researched samples were the BMI and high self-assessment of physical fitness. There was no significant variation in the level of physical activity and the amount of leisure time, both in those studying in the Visegrad states and Ukraine.

## 1. Introduction

Physical activity is widely appreciated when health and quality of life are at stake, the more so that its lack may lead to severe consequences [1,2,3,4,5,6,7,8,9]. Research shows that the majority of adult citizens in Europe (43–87%) [10], Australia (43.4%), and the United States (60%) [11] tends to be inactive or do not undertake any regular physical activity. To a large extent, they lead a sedentary lifestyle.

Accordingly, studying physical activity concerning students as future elites and probable promoters of a healthy lifestyle should take a prominent place as their attitudes toward activity as personal and social experience could be followed by others [12,13].

Meanwhile, recent research shows a decline in physical activity in societies, and the most significant regression occurs in the population of students graduating from schools and those entering universities [14,15,16,17]. Studies conducted in students from 23 countries in Central and Eastern Europe have shown that only 32% of men and 18% of women meet the WHO requirements regarding physical activity. Research in Spain also revealed that 45% of students are physically inactive, which pertains mainly to women [18]. In this context, students from the Czech Republic seem to perform much better [19].

To obtain knowledge on the state and status of physical activity in youth from different countries and continents, it is advisable to introduce the same research tool recognised by the international community. Undoubtedly, such a requirement is met by the International Physical Activity Questionnaire (IPAQ) [20], adapted for use in Poland, which allows for testing various social groups aged, i.e., 15–69 [21]. The high status of the International Physical Activity Questionnaire is best evidenced by the fact that it has been validated in many countries on different continents and recommended by international researchers’ milieus [22,23,24,25,26]. This tool has been utilised to assess the physical activity of students in many European countries including Ukraine [27], Croatia [28], Turkey [29], France [30], Poland [31,32,33] whereas in other continents—Taiwan, the United States, South Africa [34], and India [35]. For example, when used in South Africa, it revealed unfavourable outcomes. The mean value of MET in both white- and black-skinned individuals was 3002 MET in men and only 1754 MET in women, which Bloemhoff [36] referred to as ‘epidemics of physical inactivity’.

The great value of the so far conducted research on physical activity in students is that it was carried out using the same tool (IPAQ) in different fields of study [37,38,39].

It is also valued in Eastern European countries, with their common experience of transition from one economic and political system into another. Joining the European Union has affected the populations of the new member states of the European Commonwealth.

Accordingly, a question arises how the recent admission of the Visegrad countries to the European Union has affected their PA level and what changes, in the light of current research, have been visible in Ukrainian physical activity once it separated from the former Soviet bloc in 1991. The group chosen to conduct such analysis are students. It seems worth examining closer their lifestyle, including physical activity, while functioning in the new socio-economic environment. The aim of the study was to assess physical activity and factors that determine it in students from different European countries to compare and contrast PA level.

## 2. Material and Methods

### 2.1. Design and Pparticipants

The research was conducted in the years 2013–2016 and comprised 5008 males and females, including 2237 (55.3%) students from Ukraine from Eastern European National University, Lutsk, and Ternopil State Medical University and 2237 (44.7%) from the Visegrad countries, i.e., University of Pécs in Hungary, University in Kosice, Slovakia, University of Ołomuniec in the Czech Republic and the State School of Higher Education in Biała Podlaska with a mean age of 20.5 ± 2.1 years. A more detailed profile of the respondents’ characteristics is presented in Table 1.

A particularly interesting issue was to identify factors determining the PA level in the group of the examined countries.

### 2.2. Instruments

The International Physical Activity Questionnaire (IPAQ) in its extended version assessing four domains of activity, which was supplemented with original questions regarding perceived physical fitness (high, moderate, low), available free time (sufficient, too little, insufficient amount), as well as body mass and height. The research did not require the bioethical commission consent.

### 2.3. Procedure

To recruit students, there was selected one university from the four Visegrad countries and two from Ukraine, which allowed for guaranteeing that the populations of the respondents from both tested groups are similar. Formerly, the permission was obtained from the university authorities and students participating in the research. Due to some incomplete data, the data provided by 160 students from four Visegrad countries and 59 from Ukraine were eliminated from the final computations. The students completed the IPAQ questionnaire via the Internet in the INDARES system in groups of 15–20 persons in computer labs at their universities. The authors of the article participated in the conducted research together with some trained assistants and IT specialists.

The “face to face” data collection allowed for asking questions on an individual basis, which guaranteed that the answers were more objective. The credibility of the IPAQ test was checked prior to the start of the research activities during the “Physical and recreational activity as well as diet of young people from V4 countries” project. The IPAQ questionnaire was supplemented with three original questions concerning body mass and height, which allowed for calculating the BMI index, as well as self-perceived physical fitness (high, moderate, low) and the amount of free time (sufficient, too small, no time).

### 2.4. Analysis of Data

The statistical analysis was performed using the STATISTICA v 10 programme. The level of total physical activity and its areas was presented in arithmetic means and standard deviations. In order to detect statistically significant differences between the surveyed students from Ukraine and the Visegrad countries, the non-parametric Mann-Whitney test was used as it does not comply with the normal distribution assumptions. Statistically significant differences between Ukrainian and Visegrad students were also sought for such variables as men, women, underweight persons, BMI values and overweight, as well as respondents’ perceived physical fitness (low, medium and high) and the amount of leisure time (insufficient, sufficient and no free time). As for the comparison of BMI, self-assessment of physical fitness and the amount of available time in the surveyed students from Ukraine and the Visegrad countries, the findings were presented in per cent using the Pearson Chi square test. In all the analysed cases, the significance level was assumed at *p* = 0.05.

## 3. Results

### 3.1. Total Physical Activity and its Areas (Domains)

Those who study in the Visegrad countries demonstrate a significantly higher level of overall physical activity compared to their counterparts in Ukraine. Similar patterns have been shown in the areas of physical activity at work- and school-related activity, transportation and home domains. Only in sports, there was no significant difference (Table 2).

### 3.2. Total Physical Activity in Students and Gender

Male students in the Visegrad countries demonstrate a higher level of total physical activity as well as work and study-related activity. No significant differences were found in other areas.

As for the women from the Visegrad countries, significantly higher values were visible in total physical activity as well as three other areas, i.e., work and study-related, transportation and housework activity. Similarly, to men, there were no significant differences in sports activity in students representing those states (Table 3).

### 3.3. BMI Indicator

**Figure 1:**
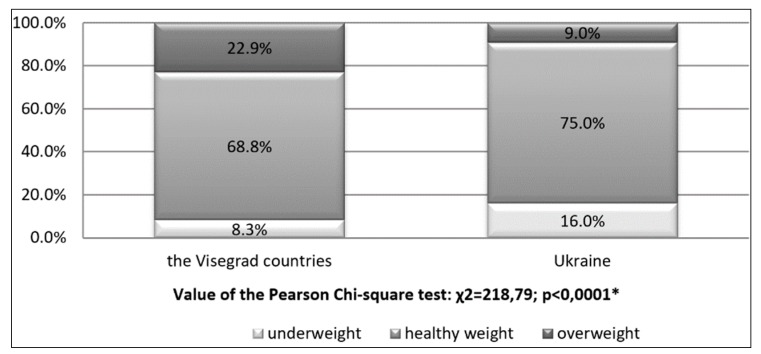
BMI classification in various countries. * Significant variation at *p* < 0.05.

The BMI index classification differs significantly between the analysed countries in favour of the students coming from Ukraine. The healthy BMI value was demonstrated in 75% of the student population as compared to 68.8% in the Visegrad countries. Being overweight was evidenced only in 9% of the Ukrainian youth, whereas in the Visegrad countries in as many as 22.9% students (Figure 1).

### 3.4. Total Physical Activity, its Areas and the BMI Index

The persons who were underweight and exhibited proper BMI index in the Visegrad countries were involved in significantly higher amount of physical activity (totally) compared to their peers from Ukraine, respectively 5666 MET and 3840 MET. The same positive trends were visible in students with healthy BMI, respectively—5721 MET and 4286 MET. However, no significant difference was found with regard to the overweight persons (Table 4).

### 3.5. Self-Assessment of Physical Fitness

**Figure 2:**
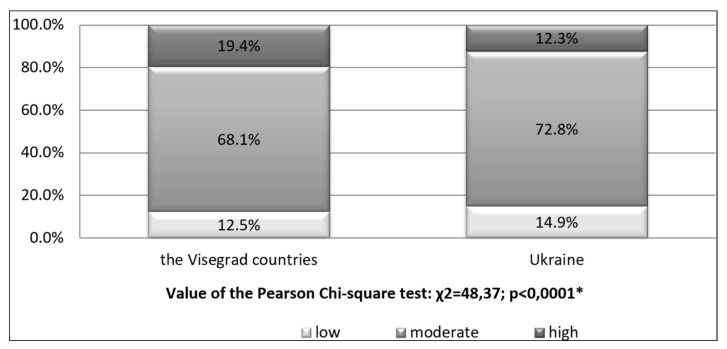
Self-assessment of physical fitness in different countries. * Significant variation at *p* < 0.05.

Self-assessment of physical fitness significantly differentiates the examined students favouring those from the Visegrad countries, who assess is as low—12.5%, moderate—68.1%, or high—19.4%. The Ukrainian findings revealed the following values: 14.9%, 71.8% and 12.3% (Figure 2).

### 3.6. Total Physical Activity, its Areas and Self-Assessment of Physical Fitness

As for self-assessment of own efficiency at the moderate and high level, the respondents from the Visegrad countries demonstrated a higher level of total physical activity than those in Ukraine. There were no significant differences perceived in the self-evaluation of physical fitness in those who viewed their activity as low (Table 5).

### 3.7. Free Time

**Figure 3:**
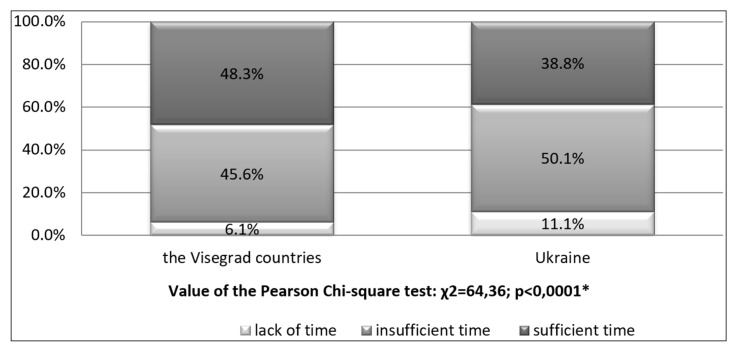
Amount of leisure-time in different countries. * Significant variation at *p* < 0.05.

The amount of the available free time varied significantly in students from the analysed countries. It is youth of the Visegrad countries who enjoy more of leisure time (48.3%) than their peers from Ukraine (38.8%). Too little amount was found in 45.6% and 50.1%, respectively. Furthermore, 6.1% of the respondents from the Visegrad countries and 11.1% from Ukraine point to lack of leisure time (Figure 3).

### 3.8. Total Physical Activity, its Areas and Free Time

The respondents from the Visegrad countries show significantly higher physical activity in the group of students who complain of little free time and those who have sufficient amount of it. However, no significant differences were found in those who lacked free time (Table 6).

## 4. Discussion

The comparison of the level of physical activity in students from the Visegrad countries and Ukraine may show tendencies in PA at a higher than a state level. The research can give a more complete picture of trends in other countries, which should allow for more precise assessment of their lifestyle concerning such variables as gender, self-assessment of physical fitness, free time, and BMI. The number of students involved in the study seems representative for the young populations in both examined samples although they derive entirely form one social group. Therefore, the obtained scores of the total weekly physical activity, especially in the students of the Visegrad countries (5588 MET/min/week) allow for their favourable evaluation as compared to the students tested in other counties and continents, i.e., Poland [31], Africa [36] or China [37].

Significantly lower level of physical activity was recorded in students from Ukraine (4233 MET) as compared to those in the Visegrad states. However, both in students from Ukraine and those coming from the Visegrad countries physical activity was noticeably lower in women as compared to men, respectively 4816 MET and 3821 MET in Ukraine, and 6023 MET and 5190 MET in the Visegrad countries, which is a common phenomenon in students from other countries [28,34,40,41] and other social groups [17,41]. The data concerning significantly lower activity in women than in men, with unquestionably lower in women from Ukraine in relation to the Visegrad countries, seem to point to an unfavourable phenomenon, especially that maintaining good health now and in the future is vital, the more so for women wishing to become mothers.

What turns out to be positive in the conducted research is that the parameters of the BMI index are correct especially in students from Ukraine, where a proper value was identified in 75% student population, with only 9.0% being overweight. These results contrast with significantly worse values in the Visegrad states, with their 68.8% and 22.9% scores respectively. In the Visegrad group, there were no significant differences in total physical activity regarding the BMI index, i.e., proper—5721 MET, underweight—5666 MET, and overweight—5527 MET [42,43,44]. More substantial differences were found in the group of students from Ukraine; that is, 4286 MET, 3840 MET and 4674 MET respectively.

As for the available free time, it turns out that its amount leads to significant differentiation of students in the analysed countries. The highest value was recorded in those from the Visegrad countries, i.e., 48.3% as compared to 38.8% from Ukraine. Also, the tested students from the Visegrad group, especially those with sufficient amount of time, demonstrated significantly higher physical activity, i.e., 5873 MET, whereas the lowest value was found in the group of Ukrainian students—4159 MET. Perhaps it is the more advantageous economic situation in the students of the Visegrad countries that provides more free time at their disposal and, consequently, more physical activity. Significant differences in favour of the Visegrad group were also demonstrated in the respondents with insufficient free time, which may be the determinant of their higher awareness of the role physical activity plays in a healthy lifestyle.

Furthermore, the tested groups (from V4 countries) showed significantly different self-assessment of physical fitness than their Ukrainian peers, with respectively 19.4% and 12.3% estimating it as high in both populations, and 12.5% and 14.9%—as low.

More favourable (high) self-assessment of own physical fitness was correlated to the highest physical activity both in the Visegrad group (7764 MET) and Ukrainian one (4973 MET). Still, a significantly higher overall level of physical activity was visible in the Visegrad group, which was also true of those with moderate and low self-assessment, where much higher values were recorded. It can therefore be stated that people exhibiting high physical activity aptly assess their physical fitness. Significant differences in physical activity in the presented group of countries have been demonstrated in favour of the Visegrad students in the self-assessment of fitness—high and moderate levels, but no such differences were visible in those with low self-assessment of physical fitness.

These data showed that significantly higher values of physical activity were observed in V4 students in the group with the healthy BMI index although it was students from Ukraine who achieve better indicators in the BMI index. The obtained data concerning a higher level of physical activity in students of the Visegrad countries may imply that it results from several years of functioning in more favourable socio-economic conditions in the European Union. Perhaps the less favourable values of the BMI index are an indicator of a higher comfort of life in the Visegrad countries and less healthy nutrition habits, which are more typical of countries with a higher standard of living, an example of which are the inhabitants of the United States.

Some limitations of the research should be considered as well. Although, the number of students involved in the study seems representative for student populations in both countries, it must be admitted that they derive mainly from institutions that are located in regional or provincial cities, not the capitals. The prospective investigations should cover some more varied samples within each state, i.e., students from prestigious universities as well as community colleges. It is because youth residing in urban environments may greatly improve their lifestyle by easier access to events and activities, and the quality of life and opportunities as not the same as in small towns. Also, their activity regarding the transportation domain definitely shows different indicators. More varied samples, with the same chosen variables, might guarantee a more complete picture of the youth coming from universities and colleges in big and small academic centres.

Furthermore, the gathered data could be used in some comparative studies concerning youth in different countries. Here, the data on students who are usually more active and mobile than their peers on the whole might be compared and contrasted with the ones on other young people, be it working youth or unemployed. Such research might more precisely point to contemporary trends and challenges facing present and future generations. Accordingly, prospective research could bring more essential information enabling further domestic and international comparisons to be used in public health campaigns or programmes aimed at promoting healthy lifestyle.

Finally, it should be remembered that one of the factors determining students’ physical activity is the field of study and its specificity [37,38,39]. These studies show that it is worth indicating which field of study is examined, be it IT, medical courses, physical education or any other, when assessing students’ physical activity.

## 5. Conclusions

The Visegrad students demonstrate a higher level of total physical activity than Ukrainians, which shows them in a positive light as compared to others described in international studies. What seems unfavorable is a significantly lower level of total activity in women than men in both studied groups. Such a situation requires new considerations regarding programmes of physical activities targeted at women students. What appears positive about the examined students is that a vast majority of them demonstrate a healthy BMI with significantly better values achieved by Ukrainians. Healthy BMI value, high self-assessment of physical fitness as well as sufficient amount of free time in the case of the Visegrad Group should be mentioned as factors significantly determining physical activity in both groups of students.

## Figures and Tables

**Table 1:** Characteristics of the surveyed students from the Visegrad group and Ukraine.

Sample Groups		Ukraine		Visegrad Countries	
		*n*	%	*n*	%
Country	Czech Republic	-	-	503	22.5
	Poland	-	-	727	32.5
	Slovakia	-	-	512	22.9
	Hungary	-	-	495	22.1
	Ukraine	2771	100.0	-	-
Sex	women	1624	58.6	1169	52.3
	men	1147	41.4	1068	47.7
Age	Mean	19.1		21.8	
	SD	1.3		1.8	

**Table 2:** Level of total activity and its domains by different countries (Met-min./week).

	DOMAINS PA									
	Total PA		Job-Related PA		Transportation PA		Housework PA		Sport and Leisure-Time PA	
	Mean	SD	Mean	SD	Mean	SD	Mean	SD	Mean	SD
**A**	5588.5	5332.3	1689.6	2595.2	1122.6	1545.7	1131.9	1499.9	1644.4	2059.6
**B**	4233.4	3434.4	945.0	1692.0	860.2	1123.2	1096.1	1208.1	1332.1	1510.3
**TEST**	Z = 5.58; *p* < 0.0001 *		Z = 5.54; *p* < 0.0001 *		Z = 2.69; *p* = 0.0071 *		Z = 3.63; *p* = 0.0003 *		Z = 0.83; *p* = 0.4091	

Z—value of the Mann-Whitney U test; * significant variation at *p* < 0.05; A—the Visegrad countries; B—Ukraine.

**Table 3:** Level of total activity and its domains by gender in different countries (Met-min./week).

		DOMAINS PA									
		Total PA		Job-Related PA		Transportation PA		Housework PA		Sport and Leisure-Time PA	
		Mean	SD	Mean	SD	Mean	SD	Mean	SD	Mean	SD
**Men**	A	6023.9	5727.0	1892.3	2810.6	1155.2	1618.6	1141.3	1569.5	1835.2	2185.6
	B	4816.8	3809.8	1150.3	1794.0	962.8	1277.5	1038.5	1266.6	1665.2	1742.0
	TEST	Z = 2.16; *p* = 0.0309 *		Z = 2.40; *p* = 0.0164 *		Z = 0.70; *p* = 0.4810		Z = 0.84; *p* = 0.4001		Z = 1.53; *p* = 0.1269	
**Women**	A	5190.6	4913.1	1504.5	2367.5	1092.8	1476.1	1123.3	1434.1	1470.1	1921.7
	B	3821.4	3077.6	800.1	1600.8	787.8	994.1	1136.7	1163.6	1096.8	1271.4
	TEST	Z = 5.17; *p* < 0.0001 *		Z = 4.66; *p* < 0.0001 *		Z = 2.80; *p* = 0.0052 *		Z = 3.81; *p* = 0.0001 *		Z = 1.80; *p* = 0.0723	

Z—value of the Mann-Whitney U test; * Significant variation at *p* < 0.05; A—the Visegrad countries; B—Ukraine.

**Table 4:** Level of total activity and its domains by BMI in various countries (Met-min./week).

		DOMAINS PA									
		Total PA		Job-Related PA		Transportation PA		Housework PA		Sport and Leisure-Time PA	
		Mean	SD	Mean	SD	Mean	SD	Mean	SD	Mean	SD
**Underweight**	A	5666.7	5336.4	1667.6	2438.3	1302.2	1788.1	1134.8	1505.2	1562.1	2149.3
	B	3840.9	2949.1	797.1	1561.4	796.3	1034.0	1069.7	1092.1	1177.7	1196.5
	TEST	Z = 2.51; *p* = 0.0120 *		Z = 2.80; *p* = 0.0051 *		Z = 1.73; *p* = 0.0834		Z = 0.98; *p* = 0.3282		Z = 0.51; *p* = 0.6132	
**Healthy weight**	A	5721.4	5486.8	1810.2	2759.8	1128.3	1528.7	1135.3	1536.2	1647.6	2069.6
	B	4286.8	3503.0	958.9	1691.7	868.1	1139.8	1100.4	1228.0	1359.5	1540.6
	TEST	Z = 5.20; *p* < 0.0001 *		Z = 5.16; *p* < 0.0001 *		Z = 2.67; *p* = 0.0076 *		Z = 3.26; *p* = 0.0011 *		Z = 0.46; *p* = 0.6468	
**Overweight**	A	5527.3	5014.1	1510.1	2213.1	1154.1	1554.2	1167.0	1431.4	1696.1	2063.0
	B	4674.9	3684.9	1123.1	1950.3	959.7	1159.4	1141.2	1245.5	1450.8	1771.7
	TEST	Z = 0.81; *p* = 0.4167		Z = 1.21; *p* = 0.2279		Z = 0.74; *p* = 0.4605		Z = 0.90; *p* = 0.3684		Z = 0.01; *p* = 0.9923	

Z—value of the Mann-Whitney U test; * Significant variation at *p* < 0.05; A—the Visegrad countries; B—Ukraine.

**Table 5:** Level of total activity and its domains by self-assessment of physical fitness in different countries (Met-min./week).

		DOMAINS PA									
		Total PA		Job-Related PA		Transportation PA		Housework PA		Sport and Leisure-Time PA	
		Mean	SD	Mean	SD	Mean	SD	Mean	SD	Mean	SD
**low**	A	3223.6	3296.2	862.5	1425.3	760.0	1045.7	824.9	1249.8	776.2	1297.0
	B	3372.2	3214.2	695.4	1503.6	788.0	991.2	1023.9	1200.4	865.0	1267.7
	TEST	Z = 1.46; *p* = 0.1440		Z = 1.46; *p* = 0.1441		Z = 1.37; *p* = 0.1695		Z = 3.59; *p* = 0.0003 *		Z = 2.74; *p* = 0.0061 *	
**moderate**	A	5432.1	5108.8	1647.7	2520.2	1123.2	1521.2	1175.1	1487.4	1486.1	1903.9
	B	4276.3	3431.6	953.2	1745.3	893.9	1182.6	1099.4	1197.2	1329.7	1458.6
	TEST	Z = 3.62; *p* = 0.0003 *		Z = 3.78; *p* = 0.0002 *		Z = 1.85; *p* = 0.0638		Z = 1.83; *p* = 0.0673		Z = 1.85; *p* = 0.0648	
**high**	A	7764.9	6398.9	2401.2	3246.3	1343.6	1848.5	1218.0	1691.3	2801.9	2527.9
	B	4973.9	3565.6	1154.1	1559.7	771.2	938.9	1138.4	1278.6	1910.2	1885.7
	TEST	Z = 5.50; *p* < 0.0001 *		Z = 3.32; *p* = 0.0009 *		Z = 3.19; *p* = 0.0014 *		Z = 0.90; *p* = 0.3696		Z = 4.91; *p* < 0.0001 *	

Z—value of the Mann-Whitney U test; * Significant variation at *p* < 0.05; A—the Visegrad countries; B—Ukraine.

**Table 6:** Level of total activity and its domains by the amount of available leisure-free in different countries. (Met-min./week).

		DOMAINS PA									
		Total PA		Job-Related PA		Transportation PA		Housework PA		Sport and Leisure-Time PA	
		Mean	SD	Mean	SD	Mean	SD	Mean	SD	Mean	SD
**Lack of time**	A	4791.4	5539.7	1539.0	2975.6	694.1	1004.7	1067.8	1541.0	1490.5	2167.5
	B	4404.9	3934.1	1013.4	1987.8	959.8	1135.5	1241.8	1452.1	1189.9	1536.3
	TEST	Z = 0.79; *p* = 0.4322		Z = 0.52; *p* = 0.6018		Z = 3.39; *p* = 0.0007 *		Z = 2.95; *p* = 0.0032 *		Z = 1.21; *p* = 0.2252	
**Insufficient time**	A	5438.0	5062.2	1719.8	2614.3	1039.9	1476.2	1181.1	1554.1	1497.2	1830.4
	B	4240.8	3366.0	943.6	1685.5	834.4	1058.2	1150.2	1211.7	1312.5	1507.7
	TEST	Z = 3.44; *p* = 0.0006 *		Z = 3.10; *p* = 0.0019 *		Z = 0.63; *p* = 0.5274		Z = 2.60; *p* = 0.0094 *		Z = 0.18; *p* = 0.8610	
**Sufficient time**	A	5873.1	5586.9	1693.5	2555.3	1250.1	1655.3	1106.9	1455.9	1822.6	2252.2
	B	4159.3	3385.4	911.7	1608.7	871.7	1211.3	976.3	1113.5	1399.5	1517.3
	TEST	Z = 5.02; *p* < 0.0001 *		Z = 4.52; *p* < 0.0001 *		Z = 4.20; *p* < 0.0001 *		Z = 0.47; *p* = 0.6387		Z = 0.34; *p* = 0.7312	

Z—value of the Mann-Whitney U test; * Significant variation at *p* < 0.05; A—the Visegrad countries; B—Ukraine.
